# Lightweight EEG Phase Prediction Based on Channel Attention and Spatio-Temporal Parallel Processing

**DOI:** 10.3390/brainsci16010011

**Published:** 2025-12-22

**Authors:** Shufei Duan, Yuting Yan, Qianrong Guo, Fujiang Li, Huizhi Liang

**Affiliations:** 1 College of Computer Science and Technology, Shanxi University of Electronic Science and Technology, Linfen 041000, China; 2 College of Electronic Information Engineering, Taiyuan University of Technology, Jinzhong 030600, China; 2024511160@link.tyut.edu.cn (Y.Y.); gqrstudy0718@163.com (Q.G.); lifujiang10@126.com (F.L.); 3 School of Computing, Newcastle University, Newcastle NE1 7RU, UK; huizhi.liang@newcastle.ac.uk

**Keywords:** transcranial magnetic stimulation, phase prediction, real-time, accuracy, parallel prediction model

## Abstract

**Background/Objectives:** Closed-loop phase-locked TMS aims to deliver stimulation at targeted EEG phases, but real-time phase prediction remains a practical bottleneck. Timing errors are especially harmful near peaks and troughs, where small offsets can substantially degrade phase targeting. We benchmark representative predictors and develop models that improve phase consistency while reducing peak/trough lag. **Methods:** Using the publicly available Monash University TEPs–MEPs dataset, we benchmark classical predictors (AR- and FFT-based) and recurrent baselines (LSTM, GRU). To quantify extremum-specific behavior critical for closed-loop triggering, we propose Mean Lag Time (MLT), defined as the average temporal offset between predicted and ground-truth extrema, alongside PLV, APE, MAE, and RMSE. We further propose a parallel DSC-Attention-GRU architecture combining depthwise separable convolutions for efficient multi-channel spatio-temporal feature extraction with self-attention for spatial reweighting and dependency modeling, followed by a GRU phase predictor. A lightweight SqueezeNet-Attention-GRU variant is also designed for real-time constraints. **Results:** LSTM/GRU outperform AR/FFT in capturing temporal dynamics but retain residual peak/trough lag. Across stimulation intensities and frequency bands, DSC-Attention-GRU consistently improves phase consistency and prediction accuracy and reduces extremum lag, lowering MLT from ~7.77–7.79 ms to ~7.50–7.56 ms. The lightweight variant maintains stable performance with an average 3.7% inference speedup. **Conclusions:** Explicitly optimizing extremum timing via MLT and enhancing multi-channel modeling with DSC and attention reduces peak/trough lag and improves phase-consistent prediction, supporting low-latency closed-loop phase-locked TMS.

## 1. Introduction

Transcranial magnetic stimulation (TMS), as a non-invasive neuromodulation technology, has become an important means to promote functional recovery after stroke by precisely regulating cortical excitability and neural network connectivity [1]. In addition, electroencephalogram (EEG) is a noninvasive technique for recording electrical activity in the brain. It measures electrical signals from neurons in the cerebral cortex by placing multiple electrodes on the scalp. Because the synchronous firing of neurons produces weak potential differences on the scalp surface, these potential differences can be amplified and recorded to form EEG signals [2]. In EEG studies, α waves (8–12 Hz) reflect resting-state cortical synchronization and represent alert relaxation; β waves (12–30 Hz) are associated with cognitive processing in the middle- and low-frequency bands (12–20 Hz), and high-frequency bands (20–30 Hz) are associated with high alertness and anxiety [3]. The power ratio (α/β) of the two bands can be used as a neural marker of phase prediction reliability. At the same time, the therapeutic effect of TMS is highly dependent on the precise matching of the endogenous neural activity state of the brain, especially the synchronization with the phase of EEG; the phase of neural oscillation not only carries the key information for regulating physiological activities, but also directly determines the regulation efficiency of stimulation on neuron population [4,5]. Therefore, building a closed-loop TMS system based on real-time EEG phase prediction and realizing personalized intervention of “on-demand regulation” has become the core direction of improving treatment accuracy.

The TMS-EEG stimulation protocol mainly includes open-loop stimulation with fixed preset parameters, open-loop brain state-dependent stimulation [6] with parameters designed according to brain state, and closed-loop brain state-dependent stimulation with real-time monitoring and dynamic adjustment of parameters [7,8]. At present, most research focuses on open-loop brain state-dependent stimulation [4], but endogenous brain activity is the key determinant of the stimulation effect [9,10], and phase-dependent stimulation in high-frequency bands needs millisecond loop delay and jitter control, so the research of the real-time closed-loop phase-locking method needs to be carried out urgently.

The research of EEG phase prediction has experienced an evolution process from linear modeling to nonlinear optimization, from theoretical verification to clinical application. Early research is based on linear time series models, such as autoregressive (AR) spectrum estimation and the forward prediction method proposed by Chen et al.; through fixed window fitting and short-time Fourier analysis, phase prediction of millisecond α wave and other target frequency bands is realized, and the feasibility of phase prediction in actual scenes is verified [5]. However, the non-stationarity, non-Gaussian, and complex neural dynamics of EEG signals limit the adaptability of linear models. A comparative study of Blinowska and Malinowski confirms that nonlinear models exhibit greater robustness when dealing with complex neural activity [11].

With the development of research, phase prediction gradually moves from a theoretical model to a practical closed-loop system. Zrenner et al. confirmed through experiments that the TMS intervention effect based on EEG real-time phase prediction is significantly better than random stimulation, and its core lies in accurate identification of the neuronal excitability state [12]. Mansouri F et al. further developed a fast phase prediction algorithm suitable for real-time electrical stimulation, compressing the time window of prediction and stimulation release to less than 50 ms, and promoting the engineering landing of the closed-loop system [13]. In this process, researchers continue to improve the accuracy and stability of the system by optimizing the filter delay balance (such as Hilbert transform combined with a filter bank) and quantifying the performance differences between different brain regions and frequency bands [14,15]. Although standard RNN and other recursive/regression methods have been widely used in EEG time series modeling [16], given the non-stationarity of EEG and the rhythm of complex, fast, and possible chaotic dynamics, Common RNN is prone to gradient degradation, historical information forgetting (long-term dependence on capture ability is weak), and prediction error accumulation over time [17]. The result is that it is very difficult to reliably and stably locate the peak or trough values of the EEG phase. Therefore, when used in closed-loop TMS systems that are phase-sensitive and have high real-time requirements, standard RNN models often fail to meet the multiple requirements of low latency, high precision, and high stability.

Deep learning technology, because of its complex network structure, theoretically can map arbitrary functions to solve complex nonlinear problems, and is widely applied to the field of EEG [18,19,20]. Long Short-Term Memory Networks (LSTMs) and Gated Recurrent Units (GRUs) in deep learning algorithms, with their sophisticated gating mechanisms, can effectively model complex temporal dependencies in EEG signals, including long-term and short-term dynamic features, thus significantly improving the accuracy of signal characterization [21,22]. This characteristic makes it an important theoretical basis and technical support for phase prediction algorithms. However, experiments with phase prediction by applying LSTMs and GRUs separately found that, in the time series regression task, when the model was optimized by minimizing the error loss function between the predicted value and the true value at time t, the regressor tended to select relatively conservative estimates in the input feature space to ensure prediction stability [23]. More importantly, sliding time window-based modeling strategies have inherent perceptual delays in the face of abrupt signal trends; this mechanistic flaw leads to systematic phase lags in the prediction of signal extreme points (including peaks and troughs) by LSTMs and GRUs [24]. In order to improve the accuracy of phase prediction, it is urgent to design a phase prediction framework which can reduce the prediction error.

Although the accuracy of the prediction model is very important in the study of closed-loop EEG phase prediction, the real-time constraint in practical application scenarios cannot be ignored. During the operation of TMS systems, due to the inherent delay of hardware (such as signal acquisition, filtering processing, and stimulation trigger) and the existence of data transmission delay, it is often difficult to achieve accurate synchronous regulation with the patient’s brain neural oscillation [25]. This limitation may lead to mismatching of the timing of the release of stimulation pulses with the target phase, thereby weakening the therapeutic effect of neuromodulation. Therefore, in order to realize personalized intervention based on the real-time EEG status of patients, computational tasks such as signal preprocessing, feature extraction, and phase prediction must be completed within a very short time window (usually <10 ms) [26].

Therefore, developing a real-time phase prediction algorithm that can simultaneously meet the requirements of high-precision prediction, low-delay response and lightweight calculation is not only a core technical challenge to break through the performance bottleneck of closed-loop TMS-EEG systems, but also an important breakthrough to realize the transformation of personalized neuromodulation from laboratory research to clinical application.

The main contributions of this paper are as follows:

1. Using the publicly available TEPs–MEPs dataset from Monash University, we benchmarked conventional baselines (AR, FFT) and deep sequence models (LSTM, GRU). While deep models improve global temporal modeling and mitigate error accumulation, we found that they can still exhibit pronounced temporal misalignment at phase extrema, reflecting limited fidelity in capturing abrupt local nonlinear variations. To quantify this clinically and operationally relevant behavior, we further propose Mean Lag Time (MLT), which measures the average time offset between predicted and ground-truth extrema, thereby complementing standard accuracy/synchrony metrics and enabling an application-oriented evaluation for real-time closed-loop stimulation.
2. To alleviate the prediction lag of phase extremums and better capture the rapid changes in local EEG, this study proposes the DSC-Attention-GRU model. The DSC module extracts low-redundancy and efficient spatio-temporal features, the self-focus mechanism highlights key spatial regions, and the GRU model establishes term temporal dependencies, jointly improving the accuracy of phase prediction.
3. To facilitate the deployment of embedded and clinical systems in the future, this study proposes a lightweight SqueezeNet-Attention-GRU model. While maintaining accuracy, it simplifies the feature extraction and attention modules, reduces the computational load, and provides a solution for the efficient use of closed-loop TMS, which has some clinical potential.

## 2. DataSet Introduction

In this study, we used the data of electromyography (EMG) and EEG evoked by TMS published by Biabani et al. [27] at Turner Institute of Brain and Mental Health, Monash University. Our research scope is electroencephalogram (EEG) phase prediction under TMS, that is, TEPs data. Trancranial magnetic stimulation-evoked EEG responses data focus on the correlation between brain stimulation and neural responses, while the β wave phase is directly related to neural synchrony in the exercise preparation phase [28], the α wave phase shows significant regularity in stimulation-induced cortical network dynamic adjustment [29], and phase information of these two bands can more accurately reflect the temporal correlation of “stimulation-response” in TEPs data. It provides a strong physiological signal basis for the prediction model.

**Figure 1:**
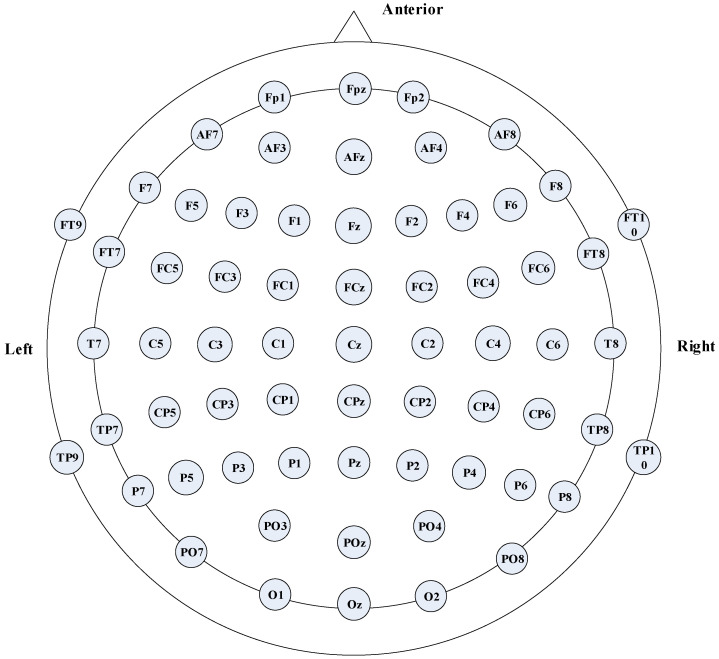
Schematic diagram of 64 EEG channels.

The TMS-evoked EEG data portion of this dataset was used in this experiment involving 20 healthy right-handed participants (6 men and 14 women, mean age 24.5 years). Biphasic TMS figure-of-eight coils and stimulators were used in the experiment. The conditioned reflex stimulation was set at 80% resting motor threshold (RMT) or 120% RMT based on RMT, and the paired pulse stimulation was carried out in combination with the suprathreshold test stimulation. EEG signals were collected using 62 Ag/AgCl electrodes in a 10−20 standard layout, electrode positions digitized by the neuronavigation system and integrated with NMRI scans, grounded AFz channels, and referenced FCz channels. The signal was amplified by a factor of 1000, low-pass filtered at DC−2000 Hz, and recorded at a sampling rate of 10 kHz. White noise and foam under coil were used to reduce interference. A schematic diagram of the 64 EEG channels involved is shown in Figure 1, including frontal, parietal, temporal, and occipital regions. Through this schematic diagram, the position of each electrode in different brain regions and the topological relationship between channels can be visually observed.

The RMT referred to in this dataset is the lowest stimulation intensity that elicits the smallest measurable motor-evoked potential when the motor cortex is stimulated by a single pulse of TMS in a state of complete muscle relaxation [30]. TMS is classified into suprathreshold and subthreshold stimulation according to the difference in stimulation intensity relative to the individual motor threshold.

- Suprathreshold stimulation intensity refers to stimulation intensity higher than the individual motor threshold, usually used to induce motor-evoked potential and other obvious neural responses. The intensity of this stimulus is sufficient to elicit measurable muscle contractions or other physiological responses.
- Subthreshold stimulus intensity refers to stimulus intensity below the individual’s motor threshold, and usually does not cause a direct motor response.

However, subthreshold stimulation can modulate cortical excitability and affect neural network activity. Suprathreshold stimulation directly elicits observable physiological responses, whereas subthreshold stimulation does not directly elicit these responses, but the intensity of this stimulus can produce potential therapeutic effects by modulating nervous system excitability [31].

In this paper, we focus on the prediction performance of the model in α and β bands under different stimulus intensities. All the EEG data of the subjects were organized in a four-dimensional structure into a tensor of 2 × 20 × 9 × 2000, corresponding, respectively, to two stimulation intensity conditions (subthreshold and subthreshold), 20 subjects, 9 key channels (C3, FC1, FC3, FC5, CP1, CP3, CP5, C1, C5), and a time series of 2000 points for each segment. This structure supports independent modeling and comparative analysis by condition, and individual and channel dimensions, providing the model with rich cross-subject and cross-condition learning capabilities.

## 3. Study on Accumulation Mechanism of Short-Term Phase Prediction Error Based on Data Stationarity Analysis

Stationarity analysis is a key prerequisite to ensure the validity of the model in EEG phase prediction research, because EEG signals are essentially non-stationary random processes, and their statistical characteristics change with time to introduce pseudo-correlation and prediction bias [32]. Traditional methods such as the AR model strictly rely on a wide stationary assumption, FFT analysis requires piecewise stationarity [33], while deep learning models such as GRUs and LSTMs can implicitly process non-stationary features, but the stationarity pretreatment can still significantly improve their training efficiency and generalization performance [34].

In this study, C3 channel EEG data related to the left primary motor cortex were se-lected because they can accurately reflect motion-related EEG activity (especially α frequency band and β frequency band phase synchronization), and have strong temporal stability and response reliability, meeting the requirements of stationarity analysis for data consistency and predictability; their phases were obtained through Hilbert transform as inputs for predicting the phases of the subsequent 500 time points. We selected traditional phase prediction algorithms AR and FFT and deep learning algorithm models LSTM and GRU—four models based on he completed Python Pytorch framework.

After adjusting parameters, the experimental parameters of each model are shown in Table 1, where order denotes order; hidden_dim specifies the number of hidden nodes in the hidden layer; this state is passed to the next unit in the sequence, and is also used for the output calculation of the current unit; pre_len indicates forward prediction of a time point; train_window specifies how many time points in the past are used to predict future data; batch_size determines the number of samples used in a training process, and lr is the learning rate.

When using an autoregressive model, ADF (Augmented Dickey–Fuller test) statis-tics, *p*-values, and critical values need to be calculated for C3 channel data to ensure that the data is approximately stationary. ADF is a classical statistical method to test the stationarity of time series data. If there is no unit root, it is a stationary series (statistical characteristics such as mean and variance do not change significantly with time), otherwise it is a non-stationary series. A *p*-value measures the statistical significance of the difference and represents the probability of observing the current data, assuming the null hypothesis holds. The critical value is the statistical threshold at which the null hypothesis is rejected. Two kinds of EEG data numbered 001 were randomly selected for data stationarity analysis. See Table 2 for results.

The ADF statistic value of EEG data from suprathreshold stimulation was −5.617, the critical value was −3.434 at the significance level of 1%, and the critical values were −2.863 and −2.568 at the significance levels of 5% and 10%, respectively. At the three significance levels, the ADF statistic value was less than the corresponding critical value and the *p*-value was very small, so the null hypothesis could be rejected, that is, the signal had unit roots, indicating that the signal was stationary. Similarly, the EEG data signal of subthreshold stimulation is also stable.

Based on the premise of signal stationarity, the experiments of single channel single subject developed by the autoregressive algorithm, Fourier transform algorithm, and time series prediction models LSTM and GRU are studied. Phase-locking value (PLV), average phase error (APE), mean absolute error (MAE), and root mean square error (RMSE) were used.

(1) $$PLV=\frac{1}{n}\left\|\sum_{i=1}^{n}e^{j(\|{\hat{\varphi }}_{i}-\varphi_{i}\|)}\right\|$$

$\varphi_{i}$ represents the phase of the real EEG, ${\hat{\varphi }}_{i}$ represents the phase of the predicted EEG, n represents the total sample size, and the phase-locking value represents the stability of the phase difference between the predicted EEG and the real EEG, with a range of 0 to 1. The closer the phase-locking value is to 1, the more stable the phase difference is, and the better the phase-locking value between the stimulus and the brain.

(2) $$APE=\sqrt{-2\ln\left(\sqrt{{\left(\frac{1}{n}\sum_{i=1}^{n}\cos\left(\varphi_{1i}-\varphi_{2i}\right)\right)}^{2}+{\left(\frac{1}{n}\sum_{i=1}^{n}\sin\left(\varphi_{1i}-\varphi_{2i}\right)\right)}^{2}}\right)}$$

$\varphi_{1i}$ represents the *i*-th value of the first group of phase data, and $\varphi_{2i}$ represents the *i*-th value of the second group of phase data. APE can directly and sensitively reflect the accuracy of phase prediction and support algorithm optimization; the value range of APE is [0, π].

(3) $$MAE=\frac{1}{n}\sum_{i=1}^{n}|{\hat{y}}_{i}-y_{i}|$$

${\hat{y}}_{i}$ is the predicted value of EEG, and $y_{i}$ is the true value of EEG. MAE can comprehensively evaluate prediction errors and stably characterize model performance due to its robustness to outliers; the value range of MAE is greater than or equal to 0.

(4) $$RMSE=\sqrt{\frac{1}{n}\sum_{i=1}^{n}{\left({\hat{y}}_{i}-y_{i}\right)}^{2}}$$

${\hat{y}}_{i}$ is the predicted value of EEG, and $y_{i}$ is the true value of EEG. RMSE can help identify model defects and evaluate its ability to capture signal energy characteristics by highlighting the effects of large errors and correlating signal energy characteristics; the value range of RMSE is greater than or equal to 0.

To quantify the temporal lag of EEG phase prediction at peaks and troughs, we define the event-wise timing difference as

(5) $$\Delta t=t_{\mathrm{pred}}-t_{\mathrm{true}}$$

where $t_{\mathrm{pred}}$ denotes the predicted time point of a target peak/trough derived from phase prediction, and $t_{\mathrm{true}}$ denotes the corresponding ground-truth time point.

Let S be the number of subjects. For subject i, the lag samples at peaks and troughs are ${\left\{\Delta t_{i,k}^{\left(p\right)}\right\}}_{k=1}^{N_{i}^{\left(p\right)}}$ and ${\left\{\Delta t_{i,k}^{\left(v\right)}\right\}}_{k=1}^{N_{i}^{\left(v\right)}}$, respectively. The mean lag at peaks and troughs is computed as

(6) $${\bar{\Delta t}}_{i}^{\left(p\right)}=\frac{1}{N_{i}^{\left(p\right)}}\sum_{k=1}^{N_{i}^{\left(p\right)}}\Delta t_{i,k}^{\left(p\right)}$$

(7) $${\bar{\Delta t}}_{i}^{\left(v\right)}=\frac{1}{N_{i}^{\left(v\right)}}\sum_{k=1}^{N_{i}^{\left(v\right)}}\Delta t_{i,k}^{\left(v\right)}$$

We then define the subject-level mean lag time as the arithmetic mean of the peak and trough mean lags:

(8) $${\bar{\Delta t}}_{i}=\frac{1}{2}\left({\bar{\Delta t}}_{i}^{\left(p\right)}+{\bar{\Delta t}}_{i}^{\left(v\right)}\right)$$

Finally, the overall mean lag time (MLT) across subjects is given by

(9) $$\mathrm{MLT}=\frac{1}{S}\sum_{i=1}^{S}{\bar{\Delta t}}_{i}=\frac{1}{2S}\sum_{i=1}^{S}\left({\bar{\Delta t}}_{i}^{\left(p\right)}+{\bar{\Delta t}}_{i}^{\left(v\right)}\right)$$

α band data of 20 subjects under suprathreshold and subthreshold stimulus intensity were input into specific models, respectively. After five repeated experiments, statistical analysis was carried out on output indicators, and their average values were recorded in Table 3 and Table 4, respectively.

Experimental results showed that GRU and LSTM consistently outperform AR and FFT in phase consistency, phase accuracy, waveform error, and peak and trough temporal alignment. Under suprathreshold α stimulation, GRU and LSTM achieve the highest PLV of 0.983, while reducing APE to 0.154 and 0.126 and lowering MAE and RMSE to 0.442 and 1.173 for GRU and 0.369 and 1.047 for LSTM, compared with 1.534 and 2.122 for AR and 2.236 and 2.644 for FFT. GRU also yields the smallest mean lag time of 7.762 ms, better than LSTM at 7.863 ms, FFT at 8.052 ms, and AR at 9.754 ms. Under subthreshold α stimulation, GRU provides the best overall results with PLV of 0.981, APE of 0.164, MAE of 0.422, RMSE of 1.132, and the lowest MLT of 7.771 ms, compared with 7.865 ms for LSTM, 8.651 ms for FFT, and 9.365 ms for AR. Although a residual lag remains, GRU reduces MLT relative to AR from 9.754 ms to 7.762 ms under suprathreshold stimulation and from 9.365 ms to 7.771 ms under subthreshold stimulation, indicating improved peak and trough alignment alongside higher phase locking and lower prediction error.

**Figure 2:**
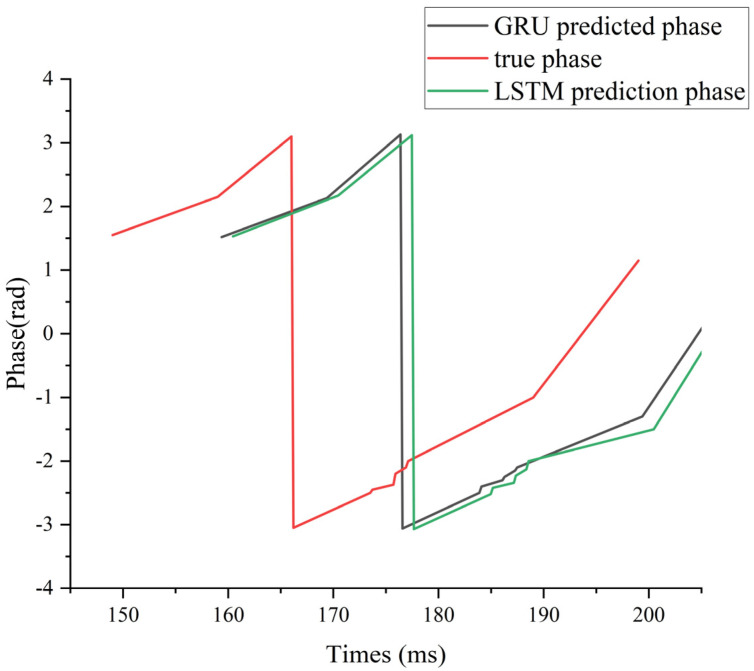
Phase prediction results of α frequency band data of subject 006 with suprathreshold stimulation intensity.

To illustrate this lag effect more intuitively, we use subject 006 under supra-α as an example. As can be seen from Figure 2, LSTM and GRU can effectively model the dynamic temporal characteristics of EEG signals through their gating mechanism, so that the model can accurately predict the phase evolution in most time segments. However, near extreme phase regions (peaks and troughs), the model often exhibits prediction lag, which is consistent with Mendonça F et al. [24]. This is mainly attributed to its insufficient ability to respond to abrupt patterns, and it is difficult to catch local changes with rapid transitions in time. The high noise, non-stationarity, and individual difference in the EEG signal itself make extreme points often accompanied by instantaneous disturbance or artifacts, which further amplifies the error and uncertainty of the model at critical moments. Therefore, although LSTM and GRU perform well in global timing modeling, they still have some limitations in accurately identifying phase extremes.

## 4. Parallel Phase Prediction Model Based on DSC-Attention-GRU

The extreme point lag phenomenon of LSTM and GRU models in EEG phase prediction stems from the inherent characteristics of their architecture and training mechanism: the optimization goal of minimizing the error at a single time makes the model over-dependent on local continuity features, which reduces the sensitivity to phase abrupt changes; the fixed length sliding time window limits the modeling ability of long-range neural oscillation dynamics, resulting in structural delay when dealing with non-stationary signals [35]. The experiment in the Section 3 of this paper also verifies this viewpoint, and this double modeling defect is the fundamental reason for the deviation between the predicted phase and the true extreme time series.

**Figure 3:**
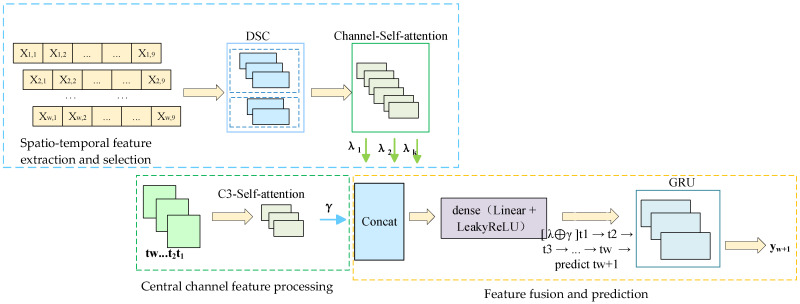
DSC-Attention-GRU parallel phase prediction model.

In order to solve the above problems, this paper constructs a DSC-Attention-GRU parallel phase prediction model, which integrates the spatial features extracted by deep separable convolution and the temporal features enhanced by the attention mechanism to realize multi-dimensional information integration, and provides an innovative solution to solve the phase prediction lag problem. The model consists of three modules: spatio-temporal feature extraction and selection, central channel feature processing, and feature fusion and prediction. In the spatial feature extraction and selection module, the spatial feature of each channel at each time point is obtained by convolution of in DSC, and the important spatial features are filtered by the self-attention mechanism. In the central channel feature processing module, the self-attention mechanism is used to interact and integrate information among elements in the sequence, and the attention weight to each element is dynamically adjusted, so as to capture the complex dependence relationship in the sequence; in the feature fusion and prediction module, the spatial features between each channel and the central channel feature are fused by using the full connection layer, and the fused features are input to GRU and the prediction result is output. To evaluate the cross-subject generalization ability of the model, this paper adopts a training/validation/testing strategy based on the random division of subjects. Specifically, all the subject data were divided into 12 training sets, three validation sets, and five test sets, which were, respectively, used for model training, hyperparameter validation, and generalization testing. There was no overlap among the subjects in each group. During the testing phase, the model performs feature extraction and prediction for each unseen subject individually and calculates its prediction performance in terms of the instantaneous phase. The generalization performance of the model in non-individualized scenarios was evaluated by the system through average performance metrics (including PLV, APE, MAE, RMSE, and MLT) on multiple independent test subjects. The model was trained with Adam and an MSE loss. The initial learning rate is 1 × 10^−5^. If the loss does not improve after 10 consecutive validations, ReduceLROnPlateau will reduce it by 10 times. Set the epoch to 400, random seed to 0, and the batch size to 32. We report model complexity in terms of the number of trainable parameters and computational cost measured by MACs/FLOPs for a single forward pass under the same input setting, together with inference latency on our hardware. The proposed DSC-Attention-GRU contains approximately 7.37M trainable parameters (about 29.5 MB in FP32), with the majority of parameters contributed by the pointwise convolution and the GRU layer, while the attention modules add a negligible number of parameters. Figure 3 represents data from channel *j* at time *i*, i∈{1,2,…,2000}, j∈{C3,C1,C5,FC1,FC3,FC5,CP1,CP3,CP5}; γ=f(Central Channel)} represents features from the central channel; $\lambda_{k}=g(\mathrm{Spatial}\;\mathrm{Features})$ represents spatial features.

### 4.1. Spatio-Temporal Feature Extraction and Screening

**Figure 4:**
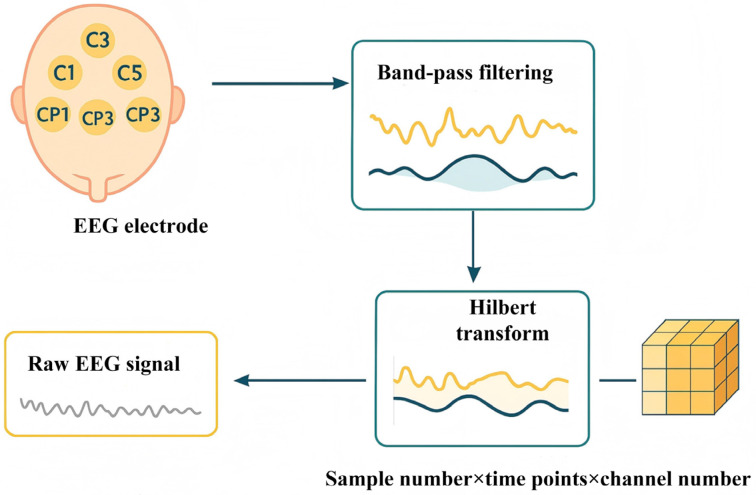
EEG signal preprocessing.

In this research, we first collected EEG signals from nine motion-related channels. To extract the phase characteristics corresponding to the α frequency band (8–13 Hz) and β frequency band (13–30 Hz) in the signal, band-pass filtering processing is first implemented on the original multi-channel EEG signal. The filter adopts the first-order IIR Butterworth structure, combined with a sampling rate of 10,000 Hz, and uses the bidirectional zero-phase filtering method to eliminate phase distortion. On this basis, the Hilbert transform is used to construct analytical signals for each channel signal after filtering, extract the phase value of the complex signal, and thereby obtain the instantaneous phase at each time point. This instantaneous phase signal is set as the supervisory target of the prediction model to guide the subsequent deep learning model in completing the time series modeling and prediction tasks of EEG phases. Ultimately, the processed data is constructed into a three-dimensional tensor of “sample number × time point × channel number”, which serves as the structured input for the deep model. The preprocessing process of the above EEG signals is shown in Figure 4.

**Figure 5:**
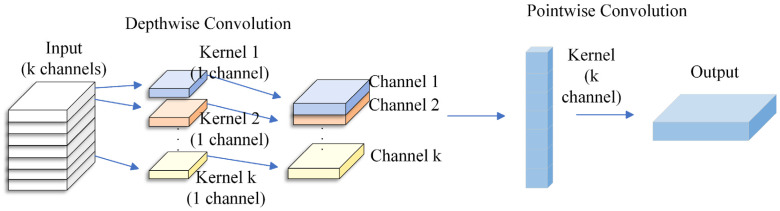
Schematic diagram of DSC structure.

This module achieves efficient extraction of spatial features through a depthwise separable convolution structure. Among them, deep convolution uses 1 × 3 convolution kernels to extract local features of each channel in the time dimension, and point convolution uses 1 × 1 convolution for channel fusion to enhance the expression ability of spatial features. After each layer of convolution, the LeakyReLU activation function is used. Enhance the nonlinear expression ability, and its structure is shown in Figure 5.

**Figure 6:**
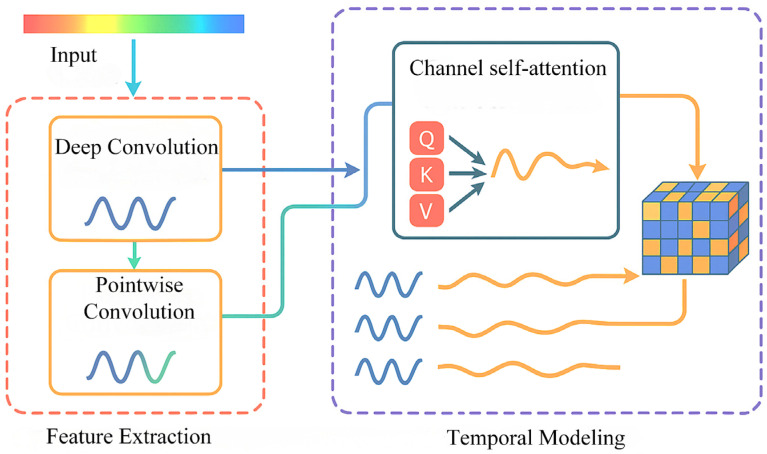
Flowchart of spatio-temporal feature extraction and screening.

Then, to capture the dynamic dependency of each channel in the time dimension, this paper defines an independent self-attention module for each channel, respectively. This module models the attention weights through three linear transformations of Query, Key, and Value, and calculates the attention distribution through Softmax normalization, thereby dynamically weighting the time series. The output of each channel is (N,T,d), where d = 10 is the hidden dimension. Eventually, the outputs of the nine channels are concatenated in the feature dimension to form (N,T,90). The flow chart is shown in Figure 6.

### 4.2. Central Channel Feature Processing

**Figure 7:**
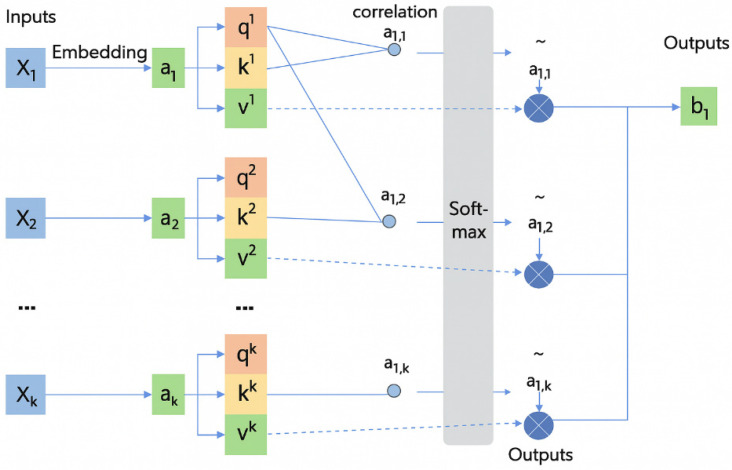
Self-attention mechanism diagram.

In addition to the multi-channel attention mechanism described above, since the C3 channel is located in the central motor cortex, its signal has the most direct correlation with actual motor planning and neural response. Therefore, designing attention paths allows the model to analyze its phase change pattern from a finer and more focused perspective. In order to further improve the modeling ability of the model for the target prediction channel (C3 channel), the system additionally sets up a self-attention branch (see Figure 7 for schematic diagram). This branch only processes the time series of the C3 channel itself, and realizes the learning of its internal time dynamic characteristics through a query-key-value mapping relationship. Its formula is expressed as follows:

(10) $$Q=X^{\left(C3\right)}W^{Q}\in {\mathbb{R}}^{B\times T\times d}$$

(11) $$K=X^{\left(C3\right)}W^{K}\in {\mathbb{R}}^{B\times T\times d}$$

(12) $$V=X^{\left(C3\right)}W^{V}\in {\mathbb{R}}^{B\times T\times d}$$

where $X^{\left(C3\right)}$ represents the shape of the C3 channel; Q represents the query vector, K represents the key vector, V represents the numerical vector, and $W^{Q},W^{K},W^{V}$ is the corresponding weight matrix; B is the number of samples tested, *T* is the time step, *d* = 10, and is the set attention dimension. A weighted feature representation is then generated for each time step by attention weight calculation to highlight signal contributions at important moments (peaks/troughs):

(13) $$\mathrm{Attention}(Q,K,V)=\mathrm{Softmax}\left(\frac{QK^{⊤}}{\sqrt{d}}\right)V$$

where $\frac{QK^{⊤}}{\sqrt{d}}$ represents the similarity between each pair of time points; normalized weights are obtained after Softmax; and finally, the output $Z^{\left(C3\right)}\in {\mathbb{R}}^{B\times T\times d}$ is obtained by the weighting and summing value with these weights. Where $Z^{\left(C3\right)}$ is the C3 channel output with time-weighted features.

### 4.3. Feature Fusion and Prediction

After feature extraction and fusion, the model concatenates the channel attention output $Z^{\left(\mathrm{All}\right)}\in {\mathbb{R}}^{B\times T\times 90}$ and C3 self-attention output $Z^{\left(C3\right)}\in {\mathbb{R}}^{B\times T\times d}$ in the feature dimension to form a unified fusion feature vector:

(14) $$Z^{\mathrm{fused}}=\left[Z^{\left(\mathrm{All}\right)}∥Z^{\left(C3\right)}\right]\in {\mathbb{R}}^{B\times T\times 100}$$

and the dimension compression is carried out through a fully connected layer containing a LeakyReLU activation function, so that different source information can be combined and transformed linearly and nonlinearly in a common space, so as to form a compact feature representation suitable for time series modeling:

(15) A=LeakyReLU(XW+b)

where $A\in {\mathbb{R}}^{n\times k}$ is the compressed compact feature representation, n is the time step, d is the feature dimension, X is the input data, W is the weight matrix, and b is the bias term. In order to further eliminate the differences in data distribution among subjects, the fusion features are standardized to make their mean zero and variance one, effectively improving the generalization ability and training stability of the model.

Ultimately, the fused and standardized features are input into the single-layer GRU neural network. The model uses a sliding window mechanism (with the window length set to 48) to construct training samples, with an input dimension of 64 and the number of hidden units set to 1024. Meanwhile, a dropout coefficient of 0.2 is introduced to suppress overfitting. By capturing the evolution process of the feature state within the current time window, the prediction of the EEG phase is ultimately achieved.

### 4.4. Validation of Model Validity

In terms of model validity verification, six differential models were constructed based on the central channel and the surrounding eight channel data of the subjects, and the multi-feature extraction components such as DSC and the self-attention mechanism were fused, and the time series modeling units such as LSTM, GRU, and WavNet were combined. This was to verify the effectiveness of the module we selected and ensure that the experimental detail designs of other models are consistent with DSC-Attention-GRU. The ablation experiment was designed to explore the performance of each model module combination in the EEG phase prediction task systematically by using different stimulation intensity and EEG frequency band data, so as to provide a multi-dimensional and refined experimental basis for screening the optimal prediction model.

In this process, the performance of six models in terms of subthreshold stimulation α frequency band data was examined. The specific results are presented in Table 5.

The PLV of both the DSC-LSTM and DSC-Attention-GRU models are higher than those of the C3-Attention-LSTM model. This indicates that the DSC module can effectively reduce feature redundancy and suppress noise interference. While reducing the MAE and RMSE, the performance of the model in feature semantic recognition and noise resistance is improved, enhancing the robustness of the model.

From the perspective of model performance comparison, the comprehensive performance of DSC-LSTM is superior to that of DSC-Attention-LSTM. Analyzing the reasons, although the signal in this task scenario is relatively stable, the proportion of background noise is relatively high. At this time, the attention mechanism not only fails to function to improve the model performance, but also increases the model complexity by introducing additional parameter calculations, while amplifying the negative impact of noise interference. In contrast, DSC-LSTM, with its simple and efficient LSTM network structure, can stably capture the phase variation patterns in such relatively simple tasks, thereby maintaining a low APE, MAE, and RMSE. It is worth noting that although the DSC-Attention-LSTM model achieved a relatively high PLV, its prediction accuracy did not improve simultaneously. Instead, it may experience a decline in overall performance due to improper distribution of attention weights and excessive focus on local features. This also confirms that the attention mechanism is more suitable for the precise capture of local detail features. The cross-model comparison results further indicate that the synergistic effect of the DSC module and the attention mechanism can more accurately fit the dynamic patterns of complex time series. Among them, the DSC-Attention-GRU model achieves the optimal balance between PLV and error control, and its anti-noise ability is particularly outstanding. Relying on the parameter simplification advantage of the GRU architecture, combined with the feature purification of DSC and the detail-focusing ability of attention, this model ultimately achieves a dual optimization of prediction accuracy and computational efficiency. DSC-Attention-GRU consistently reduces the mean peak/trough timing lag by 0.21–0.25 ms (2.7–3.2%) relative to the baselines, indicating improved peak/trough temporal alignment and alleviated phase-lag effects.

In summary, the DSC module lays a high-quality feature foundation for the model, the attention mechanism enhances the ability to capture local key features, and the GRU architecture ensures the efficiency of time series modeling. The organic integration of the three makes the DSC-Attention-GRU model slightly superior to other comparison models in terms of prediction accuracy, robustness, and efficiency.

When the EEG signals were subjected to suprathreshold stimulation intensity (i.e., stimulation intensity exceeding threshold), the evoked EEG signals were analyzed. The performance of the six models in α frequency band data is shown in Table 6.

The APE of DSC-Attention-LSTM is 13% lower than that of Attention-LSTM, indicating that DSC can improve the prediction accuracy. The APE of DSC-LSTM is 44.8% lower than that of C3-Attention-LSTM (without DSC), indicating that DSC can reduce error by optimizing feature representation. The PLV of the model with DSC is close to 1.000, which shows that DSC enhances the robustness of the model and reduces the fluctuation. APE and MAE of DSC-Attention-LSTM decreased by 85.3% and 83.4%, respectively, compared with DSC-LSTM, indicating that the attention mechanism can reduce redundant information interference. DSC-Attention-GRU is optimal on all error metrics, significantly lower than DSC-Attention-LSTM on multiple metrics, and lower than DSC-Attention-WavNet on MAE and RMSE, proving that the GRU architecture combined with DSC and Attention is more suitable for tasks. GRU is a lightweight, improved version of LSTM with low computational complexity and the ability to model long sequence dependencies. DSC-Attention-GRU has high precision, high efficiency, and strong generalization, and has significant advantages in time series prediction. DSC-Attention-GRU consistently reduces the mean peak and trough timing lag by 0.20–0.23 ms (2.6–2.9%) compared with the baselines, indicating improved peak and trough temporal alignment and alleviated phase-lag effects.

When the EEG signals were subjected to subthreshold stimulus intensity, the evoked EEG signals were analyzed. The performance of the six models in β frequency band data is shown in Table 7.

Comparing the PLV of DSC-Attention-LSTM and C3-Attention-LSTM, the PLV of the model with DSC is full score, and the PLV of the model without DSC is slightly lower, which indicates that DSC is very important to improve the stability of the basic performance of the model. Comparing the PLV of DSC LSTM and Attention LSTM, they are the same, but the error index of DSC LSTM is higher than DSC Attention LSTM, indicating that DSC needs attention to achieve the optimal effect. Compared with DSC-LSTM, APE, MAE, and RMSE decreased by 69.0%, 18.0%, and 22.8%, respectively, after attention was introduced, which proved that attention could improve the prediction accuracy. DSC-Attention-WavNet can reduce the error significantly compared with DSC-LSTM under different underlying networks, indicating that its effect is universal. DSC-Attention-GRU keeps the PLV full score, while the error index is leading in an all-round way. GRU realizes a balance between accuracy and efficiency, which is the optimal scheme for multi-component cooperation. DSC-Attention-GRU consistently reduces the mean peak/trough timing lag by approximately 0.21–0.23 ms (about 2.7–2.9%) compared with the baseline methods, indicating improved temporal alignment of peaks and troughs and thus an alleviated phase-lag effect.

When the EEG signals were subjected to suprathreshold stimulation intensity, the evoked EEG signals were analyzed. The performance of the above six models in terms of β frequency band data is shown in Table 8.

Compared with DSC-LSTM, PLV increased from 0.9988 to 0.9996, and RMSE decreased from 0.0490 to 0.0294, indicating that the attention mechanism can optimize the extraction ability of important information by dynamic weight allocation. DSC-Attention-GRU is superior to DSC-Attention-LSTM and other baseline models. It inherits the core advantages of DSC-Attention and realizes a balance between accuracy and efficiency by using the high efficiency of GRU. In terms of temporal alignment, DSC-Attention-GRU achieves the lowest mean lag time (MLT = 7.5026 ms), reducing the peak/trough lag by 0.26–0.28 ms (3.4–3.6%) compared with the LSTM- and WavNet-based baselines (MLT ≈ 7.77–7.79 ms), indicating improved peak/trough timing alignment and alleviated phase-lag effects.

#### 4.4.1. Statistical Analysis

**Figure 8:**
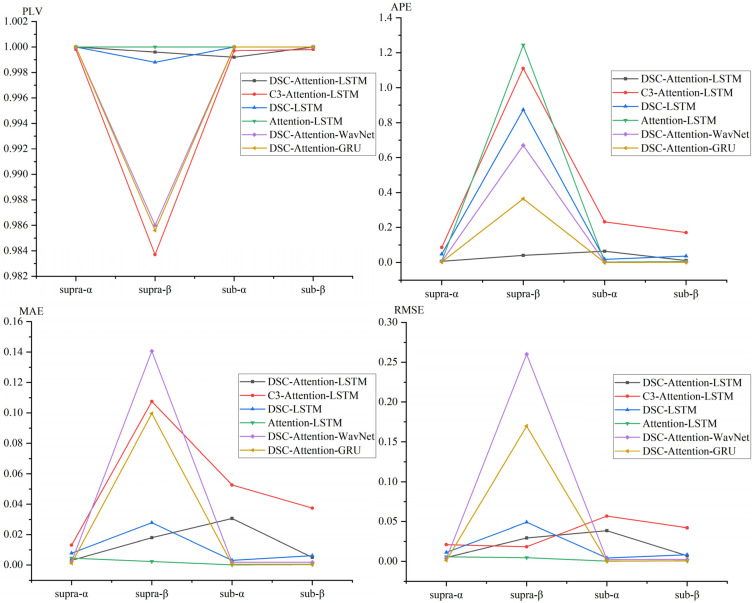
Performance comparison of six models in EEG phase prediction across four neural conditions (supra-α, supra-β, sub-α, sub-β), evaluated using four metrics.

We evaluated EEG phase prediction under TMS across four experimental conditions defined by stimulation intensity and frequency band, using PLV, APE, MAE, and RMSE to compare six deep learning models and visualize performance trends, as shown in Figure 8.

The figure shows that DSC-Attention-GRU achieved the best and most consistent performance across nearly all conditions, typically producing the lowest APE and MAE and maintaining PLV values close to 1, indicating accurate and stable phase tracking. In supra-α and sub-α, its APE was near zero with PLV close to 1, and even in the more challenging supra-β condition it remained stable, with only modest RMSE increases while keeping PLV above 0.98. In contrast, several baselines, such as C3-Attention-LSTM and Attention-LSTM, showed pronounced performance degradation in higher-frequency conditions, with APE exceeding 1.0 and PLV dropping substantially. Collectively, these results indicate that DSC-Attention-GRU offers stronger robustness and generalizability across stimulation states, supporting its suitability for real-time closed-loop TMS applications requiring reliable EEG phase tracking.

**Figure 9:**
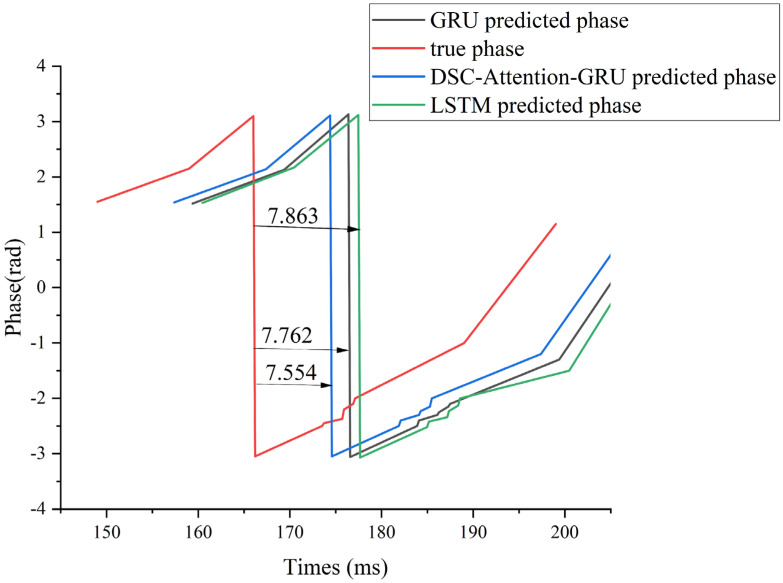
Comparison results of peak-trough value lag amounts of each model.

To illustrate this lag effect more intuitively, we use subject 006 under supra-α as an example. We mainly compared the phase hysteresis analysis of GRU, LSTM, and DSC-Attention-GRU with the real phase curve, as shown in Figure 9.

Compared with the true phase, the DSC-Attention-GRU model lags behind by an average of 7.554 ms in peak-trough value prediction. This lag is relatively small and can more accurately track the changes in the actual phase, especially at moments when the phase changes sharply. In contrast, the average lag of GRU is 7.762 ms, and that of LSTM is 7.863 ms. The lag of both is slightly greater than that of DSC-Attention-GRU. Especially when dealing with rapidly changing stages, they cannot capture phase transitions in a timely manner like DSC-Attention-GRU. Overall, DSC-Attention-GRU effectively enhances its time series modeling capability by integrating depth-separable convolution and self-attention mechanisms. It can reduce lag and be more sensitive to dynamic responses, thereby achieving higher peak-trough prediction accuracy and stronger real-time prediction capabilities.

#### 4.4.2. Comparison Experiment

To verify the effectiveness of the DSC-Attention-GRU model proposed in this paper, the EEG Phase Prediction Network (EPN) was selected as the comparison model in this study. EPN is a model specifically designed for EEG phase prediction tasks and can be directly applied to closed-loop neural regulation systems, especially suitable for the combined experimental paradigm of TMS-EEG. The core objective of this model lies in enhancing the accuracy of EEG phase prediction. The core requirement of the TMS-EEG closed-loop system is precisely to accurately select the timing of TMS stimulation based on the real-time predicted EEG phase. Therefore, EPN can efficiently predict the instantaneous phase of EEG signals, and provide key technical support for the timing regulation of TMS stimulation. The specific results are shown in Table 9.

These results indicate that, although EPN is specifically designed for online EEG phase prediction, our architecture (which combines depthwise convolution, channel-wise self-attention, and GRU-based temporal modeling) provides better phase prediction accuracy and stability across subjects in the present TMS-EEG setting. DSC-Attention-GRU consistently achieves lower MLT than EPN across all stimulation intensities and bands (e.g., 7.5026 vs 7.5761 ms in supra-β), indicating improved peak/trough temporal alignment and alleviated phase-lag effects.

## 5. SqueezeNet-Attention-GRU Parallel Prediction Lightweight Model

### 5.1. Spatio-Temporal Feature Extraction Based on SqueezeNet

Due to the remarkable advantages of the DSC-Attention-GRU parallel prediction model in capturing local and global spatio-temporal features of time series data, its prediction accuracy for complex time series data is significantly improved. However, such models currently face dual challenges: on the one hand, the large number of parameters associated with complex network architectures leads to high memory consumption, which is limited by actual computing power and storage resources; on the other hand, clinical application scenarios such as transcranial magnetic stimulation put forward stringent low latency requirements for system response. Therefore, it is urgent to carry out lightweight combination model research to balance prediction performance and computational efficiency.

**Figure 10:**
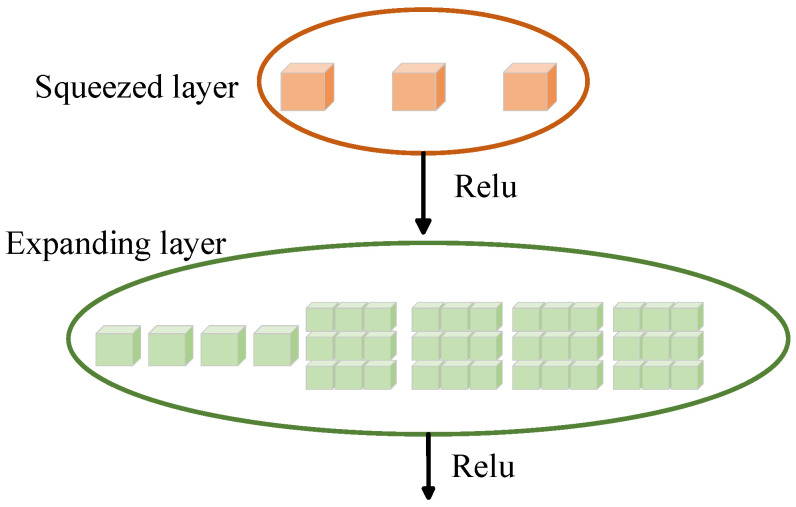
Schematic diagram of the Fire Module.

This experiment attempts to make lightweight improvements to the model by introducing the SqueezeNet model. The schematic diagram of the main Fire Module of this model is shown in Figure 10. The SqueezeNet model can effectively extract spatial features, and further use the self-attention mechanism to filter these extracted spatial features, so as to accurately select important spatial features, effectively reduce the computational complexity of the model, significantly improve the computational efficiency on the premise of ensuring prediction accuracy, and provide an efficient and reliable phase prediction solution for clinical application scenarios such as transcranial magnetic stimulation.

SqueezeNet, as a classical lightweight convolutional neural network, shows significant advantages in model size and computational efficiency. From the viewpoint of model size, the network achieves a significant reduction in the number of parameters by virtue of its unique structural design, which makes the model highly compact and effectively reduces the occupation of storage space. At the computational efficiency level, SqueezeNet uses a large number of 1 × 1 convolution kernels, which significantly reduces the computational effort and thus improves the reasoning speed of the network.

**Figure 11:**
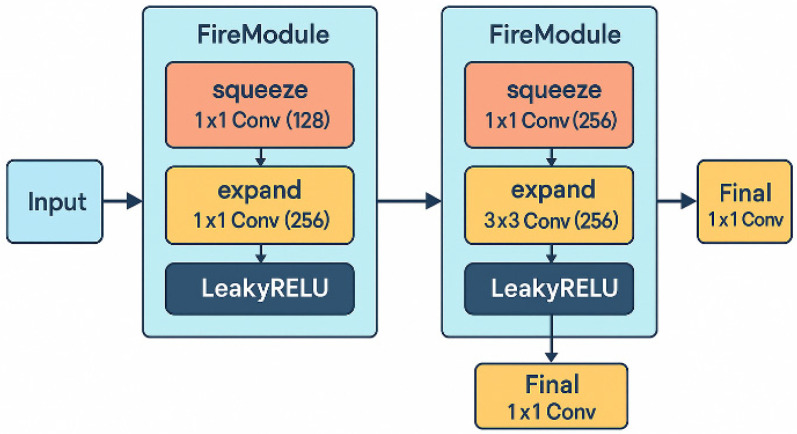
SqueezeNet network structure.

This model achieves compression by means of modular convolution Fire Module, and its core lies in reducing the number of parameters to achieve this goal. The SqueezeNet network structure adopted in this paper is shown in Figure 11.

The squeeze layer of each Fire Module uses convolution to perform channel compression operations, mapping the high-dimensional inputs of the raw EEG signal to a lower-dimensional embedding space, acting as an “information concentrator.” This not only reduces the amount of subsequent calculations, but also helps to suppress local interference. The next expansion layer uses convolution collations to re-expand the compressed features in both channel and space, thereby extracting local combined features and spatial patterns between channels in the EEG signal.

The LeakyReLU activation function is introduced to alleviate the low amplitude suppression problem in EEG signals, so that the model can retain some signal changes when dealing with near zero or negative bands, and maintain nonlinear expression ability. In addition, the final 1 × 1 convolutional layer at the back of each module further compresses or unifies high-dimensional features to prepare compact and efficient representations for subsequent processes such as attention mechanisms, feature fusion, or temporal modeling.

Finally, after the spatial modeling of the serial Fire Module, the output feature tensor will carry the structural coordination information of EEG channels under the same time window and serve as the input of the attention mechanism or GRU temporal modeling to further capture the phase evolution over time.

### 5.2. Contrast Experiment

During the experiment, data with different stimulus intensities and data covering different EEG bands were used to analyze the performance of the model under different conditions from multiple dimensions. Based on the central channel and surrounding eight-channel data, the model adopts DSC, the SqueezeNet convolutional network, and the standard attention mechanism to extract spatial features efficiently through customized convolution parameters, which provides a theoretical and practical basis for high accuracy and low computational cost EEG phase prediction.

In order to better evaluate the prediction effect of the model and its calculation speed, in addition to the five indicators used in the above experiments (PLV, APE, MAE, RMSE, MLT), a new indicator inference speed (Frames Per Second, FPS) is added in this section, indicating how many times the model can reason per second.

(16) $$FPS=\frac{Total\;\mathrm{Inferences}}{Total\;\mathrm{Inference}\;\mathrm{Time}}$$

Total Inferences represents the total number of inferences, i.e., the total number of samples run by the model, specifically the number of sequences in this experiment. Total Inference Time represents the total inference time, which is the total time it took the model to complete inference on these samples. Therefore, a higher FPS indicates a faster inference speed for the model.

This experiment was conducted on a cloud instance rented via AutoDL, operated by Shituo Cloud (Nanjing) Technology Co., Ltd. (Nanjing, China), equipped with an NVIDIA RTX 4090D GPU and a 16-core vCPU (Intel^®^ Xeon^®^ Platinum 8481C). The software environment included Ubuntu 20.04, Python 3.8, PyTorch 1.10.0, and CUDA 11.3.

In order to verify the real-time performance of the model and the accuracy of prediction more effectively, α and β frequency band data induced by suprathreshold stimulus intensity and subthreshold stimulus intensity are still used. The average results of five repeated experiments are shown in Table 10.

The table data show that the FPS index of the SqueezeNet-Attention-GRU model is better than that of DSC-Attention-GRU, the processing speed of subthreshold stimulus β frequency band reaches 1537.66 FPS, which is 8% higher than that of DSC-Attention-GRU model, and the APE, MAE, and RMSE of the subthreshold stimulus β frequency band decrease significantly by 28.8%, 14.9%, and 9.9%, respectively. In terms of phase synchronization, the PLV of the β frequency band was 0.9587 (higher than 0.9498 in control group), and the PLV of other bands was 1.0000. From the frequency band characteristics, the β frequency band error is higher than the α frequency band, indicating that high-frequency neural oscillation dynamics are more complex; PLV decreases slightly but is still high under suprathreshold stimulation, indicating that high-intensity stimulation has a marginal effect on phase synchronization but does not destroy the inherent law of signals.

Notably, MLT exhibits a narrow variation across stimulation intensities and frequency bands (approximately 7.50–7.56 ms), reaching its minimum under the suprathreshold β condition (about 7.50 ms). This indicates that the temporal alignment offset of both models remains stable and is largely insensitive to changes in experimental conditions. Consequently, the table provides complementary evidence from three perspectives—computational efficiency (FPS), phase synchrony and error levels (PLV, APE, MAE, RMSE), and temporal lag characteristics (MLT)—thereby offering more comprehensive support for the feasibility and potential effectiveness of the proposed approach in real-time closed-loop applications.

## 6. Discussion

This research’s result echoes Kirchhoff et al.’s [37] closed-loop EEG-TMS phase selection study based on Bayesian optimization, which achieved phase-lock accuracy of approximately 79% with 100 samples in healthy populations and improved to 87% in long-time sampling scenarios. In contrast, our deep time series prediction method does not require online search or dynamic reordering, but estimates the EEG phase in the future 20–40 ms in advance through forward modeling, which reduces computational overhead and is more suitable for the closed-loop system’s real-time requirements. In addition, similar to the online μ rhythm triggering mechanism proposed by Zrenner et al. [12], we also introduce channel-level attention modeling in the motor cortex axis region (C3) to enhance the modeling accuracy of phase peaks and troughs. This approach is also reflected in the EEG PhaseNet network architecture developed by Liu et al. [36], which provides robust prediction of future phases through a frequency-time domain joint convolutional network, further demonstrating the adaptability of the fused architecture to TMS phase control.

Methodologically, we introduce a multi-channel parallel attention mechanism to mine phase cooperative structures among electrodes. Especially in unsteady segments, the model shows better stability than RNN, CNN, or time-frequency analysis. At the same time, in order to solve the problems of high inter-subject variability and large distribution differences in EEG signals, we introduce a standardization mechanism after the feature fusion layer, so that the fluctuation amplitude of prediction error between different subjects is significantly reduced. Combined with the update gate and reset gate in GRU, the model can adaptively adjust the memory and forgetting ratio according to historical state, thus filtering transient noise disturbances while maintaining the periodic structure of α and β waves. Compared with the “post-trigger correction” strategy proposed by Chang et al. [38] in the delay analysis of closed-loop systems, this structure provides a forward compensation prediction idea and fundamentally alleviates the problem of trigger error accumulation.

From the perspective of model interpretability, the attention mechanism introduced in this paper not only enhances the phase prediction performance but also establishes an observable connection between the model output and the neurophysiological mechanism. In terms of spatial dimension, the channel-level attention is focused on the axial region of the motor cortex represented by C3 [39], which helps to identify the electrode positions that contribute most to the prediction of phase peaks and troughs, and can be further projected into an electroencephalogram topological map to reveal the structural correspondence with the μ rhythm generation region [40]. Although our study focuses on EEG phase prediction under TMS-evoked potentials, robustness and interpretability remain key challenges. Pratticò et al. [41] introduced a hybrid physics–AI framework that combines a finite-element physical model with data-driven learning to reduce spatial overfitting and improve generalization. This is conceptually relevant to our work because EEG models may also overfit to electrode-specific patterns and subject-dependent spatial distributions, limiting inter-subject transferability. Incorporating physiologically grounded priors or physics-informed constraints, such as head-model-based regularization or electrode-neighborhood smoothing, may therefore enhance spatial interpretability and cross-subject robustness for TMS–EEG phase prediction, representing a promising direction beyond purely data-driven modeling. In terms of the time dimension, the attention weights exhibit a specific aggregation trend before and after the phase extremum, indicating that the model tends to capture rhythmic dynamic processes such as the rising/falling edge of the phase rather than respond to random noise. This spatio-temporal joint attention distribution provides a preliminary path for extracting neural mechanism-related features from deep learning models in the future, and also offers theoretical support for the controllability and interpretability of closed-loop TMS systems. Although this study has achieved significant results in EEG phase prediction in healthy people, there are still limitations in the following three aspects. First, models currently assess α and β frequency bands at rest and do not cover phase dynamics under multitasking or cognitive load conditions. In this regard, EEG acquisition under a multitask paradigm will be introduced in the future, combined with a task label-guided joint modeling method to verify the adaptability of the model under complex neural states. Second, although the model can achieve stable prediction with some delay, it still faces the challenge of inference speed and hardware response in closed-loop deployment with very low system delay (<10 ms). In order to solve this problem, model pruning, quantization, and hardware acceleration schemes will be further introduced in the future, and system-level optimization will be combined to ensure the accuracy of closed-loop stimulation timing. Third, current models do not systematically assess adaptability to high-frequency bands (such as gamma waves) or inter-individual rhythm variation, and there are limitations on generalization ability. Therefore, a large-scale TMS-EEG joint database across subjects will be introduced in the future, and personalized model fine-tuning strategies will be explored to improve individual prediction stability and accuracy. In addition, in order to further improve the spatio-temporal modeling ability and neural structure interpretability of the model, the future work will also introduce the graph neural network (GNN) architecture to model the dynamic connection mode between each functional area of the cortex and enhance the structural constraint of feature integration between channels.

This study builds a deep phase prediction model based on TMS-EEG data from healthy participants, aiming to improve the accuracy and real-time performance of closed-loop TMS systems. Although the research is conducted on non-pathological populations, the findings have significant translational value for clinical applications. The phase dynamics and error propagation patterns derived from healthy individuals provide a normative baseline against which deviations in neurological disorders—such as dysarthria and stroke—can be assessed [42,43]. Furthermore, verifying the robustness and efficiency of deep learning models in healthy populations facilitates their deployment in clinical settings by mitigating the effects of inter-individual variability and pathological complexity [44]. Prior studies have emphasized that the reproducibility and interpretability of TMS-EEG biomarkers largely depend on modeling strategies established in healthy controls [45]. The Attention-GRU framework and its lightweight SqueezeNet-based variant proposed in this study lay a technical foundation for future real-time neural modulation systems in pathological contexts. Therefore, while rooted in healthy subject data, this work contributes essential algorithmic and methodological support for advancing closed-loop TMS applications in clinical neurorehabilitation.

## 7. Conclusions

This study proposes an EEG phase prediction method based on a channel attention mechanism and GRU temporal modeling, aiming to provide higher accuracy and lower latency prediction capabilities for phase locking in closed-loop transcranial magnetic stimulation systems. Compared with the traditional filtering threshold decision or fixed sliding window averaging strategy, the model integrates multi-channel spatial features and self-attention output of a critical region (C3) in structural design, and realizes a unified time series input vector through feature dimension splicing and nonlinear compression, so that local and global phase dynamic features are more fully expressed in time series modeling.

The experimental results show that this model demonstrates a significant improvement in prediction accuracy on the resting-state EEG dataset of healthy subjects, especially near the phase peaks and troughs of the α and β frequency bands, maintaining high temporal consistency and response sensitivity, which is significantly superior to traditional RNN, CNN, and pure time-frequency modeling methods.

## Figures and Tables

**Table 1:** Experimental model parameters.

Model	Parameters
AR	Order = 45
FFT	__
GRU	Hidden_dim = 350, pre_len = 1, train_window = 32, batch_size = 16, lr = 0.01
LSTM	Hidden_dim = 350, pre_len = 1, train_window = 16, batch_size = 32, lr = 0.01

**Table 2:** ADF statistics of C3 channel for subject no. 001.

Stimulus Intensity	ADF	*p*-Value	Critical Values 5%	Critical Values 10%	Critical Values 1%
Suprathreshold	−5.617	1.17 × 10^−6^	−2.863	−2.568	−3.434
Subthreshold	−6.119	8.97 × 10^−8^	−2.863	−2.568	−3.434

**Table 3:** Experimental results of suprathreshold stimulus intensity α frequency band data.

Model	PLV	APE	MAE	RMSE	MLT (ms)
AR	0.760	0.700	1.534	2.122	9.754
FFT	0.889	0.429	2.236	2.644	8.652
GRU	0.983	0.154	0.442	1.173	7.762
LSTM	0.983	0.126	0.369	1.047	7.863

**Table 4:** Experimental results of subthreshold stimulus intensity α frequency band data.

Model	PLV	APE	MAE	RMSE	MLT (ms)
AR	0.779	0.668	1.823	2.339	9.765
FFT	0.796	0.637	2.472	2.839	8.651
GRU	0.981	0.164	0.422	1.132	7.771
LSTM	0.980	0.173	0.455	1.148	7.865

**Table 5:** Comparison of performance results of different models with subthreshold stimulus intensity α frequency band data.

Model	PLV	APE	MAE	RMSE	MLT (ms)
DSC-Attention-LSTM	0.9992	0.0643	0.0307	0.0386	7.7821
C3-Attention-LSTM	0.9997	0.2319	0.0527	0.0568	7.8002
DSC-LSTM	1.0000	0.0181	0.0031	0.0042	7.7755
Attention-LSTM	1.0000	0.0028	0.0001	0.0003	7.7647
DSC-Attention-WavNet	1.0000	0.0006	0.0018	0.0022	7.7945
DSC-Attention-GRU	1.0000	0.0002	0.0005	0.0001	7.5522

**Table 6:** Comparison of model performance for α frequency band data of suprathreshold stimulus intensity.

Model	PLV	APE	MAE	RMSE	MLT (ms)
DSC-Attention-LSTM	1.0000	0.0069	0.0031	0.0048	7.7614
C3-Attention-LSTM	0.9998	0.0860	0.0131	0.0210	7.7698
DSC-LSTM	1.0000	0.0475	0.0078	0.0112	7.7642
Attention-LSTM	1.0000	0.0078	0.0045	0.0057	7.7684
DSC-Attention-WavNet	1.0000	0.0049	0.0022	0.0034	7.7855
DSC-Attention-GRU	1.0000	0.0024	0.0011	0.0013	7.5538

**Table 7:** Comparison results of performance of different models for β frequency band data of subthreshold stimulus intensity.

Model	PLV	APE	MAE	RMSE	MLT (ms)
DSC-Attention-LSTM	1.0000	0.0113	0.0050	0.0069	7.7625
C3-Attention-LSTM	0.9998	0.1702	0.0374	0.0420	7.7758
DSC-LSTM	1.0000	0.0365	0.0061	0.0083	7.7656
Attention-LSTM	1.0000	0.0052	0.0003	0.0005	7.7821
DSC-Attention-WavNet	1.0000	0.0015	0.0018	0.0023	7.7665
DSC-Attention-GRU	1.0000	0.0011	0.0005	0.0007	7.5545

**Table 8:** Comparison results of performance of different models for β frequency band data of suprathreshold stimulus intensity.

Model	PLV	APE	MAE	RMSE	MLT (ms)
DSC-Attention-LSTM	0.9996	0.0403	0.0180	0.0294	7.7658
C3-Attention-LSTM	0.9837	1.1106	0.1075	0.0183	7.7713
DSC-LSTM	0.9988	08742	0.0279	0.0490	7.7835
Attention-LSTM	1.0000	1.2443	0.0023	0.0045	7.7856
DSC-Attention-WavNet	0.9680	0.6705	0.1406	0.2601	7.7791
DSC-Attention-GRU	0.9856	0.3644	0.0996	0.1698	7.5026

**Table 9:** Comparison results of performance of two models for different stimulus intensities and bands.

Model	Stimulation Intensity and Band	PLV	APE	MAE	RMSE	MLT (ms)
DSC-Attention-GRU	Supra-α	1.0000	0.0024	0.0011	0.0013	7.5598
	Supra-β	0.9856	0.3644	0.0996	0.1698	7.5026
	Sub-α	1.0000	0.0002	0.0005	0.0001	7.5522
	Sub-β	1.0000	0.0011	0.0005	0.0007	7.5545
EPN [36]	Supra-α	1.0000	0.8821	0.0057	0.0061	7.5642
	Supra-β	0.9995	1.2041	0.0468	0.0507	7.5761
	Sub-α	1.0000	1.3993	0.0100	0.0116	7.5698
	Sub-β	0.9992	1.5987	0.0625	0.0706	7.5784

**Table 10:** Comparison results of model performance under different stimulus intensities and frequency band data.

Stimulus Intensity	Frequency Band	Model	PLV	APE	MAE	RMSE	FPS	MLT (ms)
Subthreshold	α	DSC-Attention-GRU	1.0000	0.0013	0.0003	0.0006	1341.24	7.5522
		SqueezeNet-Attention-GRU	1.0000	0.0015	0.0004	0.0005	1382.24	7.5462
Subthreshold	β	DSC-Attention-GRU	1.0000	0.0644	0.0005	0.0008	1423.81	7.5545
		SqueezeNet-Attention-GRU	1.0000	0.0121	0.0035	0.0042	1537.66	7.5422
Suprathreshold	α	DSC-Attention-GRU	1.0000	0.0072	0.0032	0.0046	1407.55	7.5598
		SqueezeNet-Attention-GRU	1.0000	0.0107	0.0031	0.0043	1430.62	7.5512
Suprathreshold	β	DSC-Attention-GRU	0.9498	0.7229	0.1600	0.2988	1322.10	7.5026
		SqueezeNet-Attention-GRU	0.9587	0.5142	0.1361	0.2692	1350.10	7.5011

## Data Availability

The data from the present experiment are publicly available at the Git Hub website: https://github.com/BMHLab/TEPs-MEPs (accessed on 18 October 2023).

## Abbreviations

The following abbreviations are used in this manuscript:

TMS	Transcranial magnetic stimulation
EEG	Electroencephalogram
AR	Autoregressive
LSTM	Long Short-Term Memory Network
GRU	Gated Recurrent Unit
TMS-EEG	Transcranial magnetic stimulation-Electroencephalogram
EMG	Electromyography
MEPs	Motor-evoked potentials
TEPs	TMS and electroencephalogram responses
RMT	Resting motor threshold
ADF	Augmented Dickey–Fuller test
PLV	Phase-locking value
APE	Average phase error
MAE	Mean absolute error
RMSE	Root mean square error
FPS	Frames Per Second
